# Author Correction: Unraveling the optoelectronic properties of CoSb_x_ intrinsic selective solar absorber towards high-temperature surfaces

**DOI:** 10.1038/s41467-024-47332-2

**Published:** 2024-04-26

**Authors:** Anastasiia Taranova, Kamran Akbar, Khabib Yusupov, Shujie You, Vincent Polewczyk, Silvia Mauri, Eleonora Balliana, Johanna Rosen, Paolo Moras, Alessandro Gradone, Vittorio Morandi, Elisa Moretti, Alberto Vomiero

**Affiliations:** 1 Department of Molecular Sciences and Nanosystems, Ca’ Foscari University of Venice, Via Torino 155, 30172 Venice, Italy; 2 Department of Physics, Chemistry and Biology (IFM), Linköping University, 581 83 Linköping, Sweden; 3 Division of Materials Science, Department of Engineering Sciences and Mathematics, Luleå University of Technology, SE-971 87 Luleå, Sweden; 4 Istituto Officina dei Materiali (IOM) - CNR, Laboratorio TASC, Area Science Park, S.S. 14 Km 163.5, Trieste, I-34149 Italy; 5 Dipartimento di Fisica, University of Trieste, via A. Valerio 2, 34127 Trieste, Italy; 6 Department of Environmental Sciences, Informatics and Statistics, Ca’ Foscari University of Venice, Scientific Campus Via Torino 155/b, 30173 Venice, Italy; 7 Istituto di Struttura della Materia (ISM) - CNR, S.S. 14 Km 163.5, Trieste, I-34149 Italy; 8 Istituto per la Microelettronica ed i Microsistemi (IMM) – CNR Sede di Bologna, via Gobetti 101, 40129 Bologna, Italy

**Keywords:** Nanoparticles, Devices for energy harvesting, Electronic properties and materials

Correction to: *Nature Communications*10.1038/s41467-023-42839-6,published online 10 November 2023

The original version of this Article incorrectly omitted Kamran Akbar and Elisa Moretti as corresponding authors. This has now been corrected in both the PDF and HTML versions of the Article.

