# Supplementary material for: Unraveling the optoelectronic properties of CoSbx intrinsic selective solar absorber towards high-temperature surfaces
Source: Nat Commun. 2023 Nov 10;14:7280. doi: 10.1038/s41467-023-42839-6 (PMC10638324; doi:10.1038/s41467-023-42839-6)
Supplement: Supplementary file 1 — Supplementary Information [file 41467_2023_42839_MOESM1_ESM.pdf]

Supplementary Information

Unraveling the Optoelectronic Properties of $CoSb_x$ based intrinsic selective solar absorber towards High-Temperature Surfaces

Anastasiia Taranova[1], Kamran Akbar[1], Khabib Yusupov[2], Shujie You[3], Vincent Polewczyk[4], Silvia Mauri[4,5], Eleonora Balliana[6], Johanna Rosen[2], Paolo Moras[7], Alessandro Gradone[8], Vittorio Morandi[8], Elisa Moretti[1], Alberto Vomiero[1,3*]

[1]Department of Molecular Sciences and Nanosystems, Ca' Foscari University of Venice, Via Torino 155, 30172 Venezia Mestre, Italy

[2]Department of Physics, Chemistry and Biology (IFM), Linköping University, 581 83 Linköping, Sweden

[3]Division of Materials Science, Department of Engineering Sciences and Mathematics, Luleå University of Technology, SE-971 87 Luleå, Sweden

[4]Istituto Officina dei Materiali (IOM) - CNR, Laboratorio TASC, Area Science Park, S.S. 14 Km 163.5, Trieste I-34149, Italy

[5]Dipartimento di Fisica, University of Trieste, via A. Valerio 2, 34127, Trieste, Italy

[6]Department of Environmental Sciences, Informatics and Statistics, Ca' Foscari University of Venice, Scientific Campus Via Torino 155/b, 30173 Venice, Italy

[7]Istituto di Struttura della Materia (ISM) - CNR, S.S. 14 Km 163.5, Trieste I-34149, Italy

[8]Istituto per la Microelettronica ed i Microsistemi (IMM) – CNR Sede di Bologna, via Gobetti 101, 40129 Bologna, Italy

*Correspondence: alberto.vomiero@ltu.se

This file includes:

Supplementary Figures 1-11

Supplementary Notes 9

Supplementary Tables 2

Supplementary References

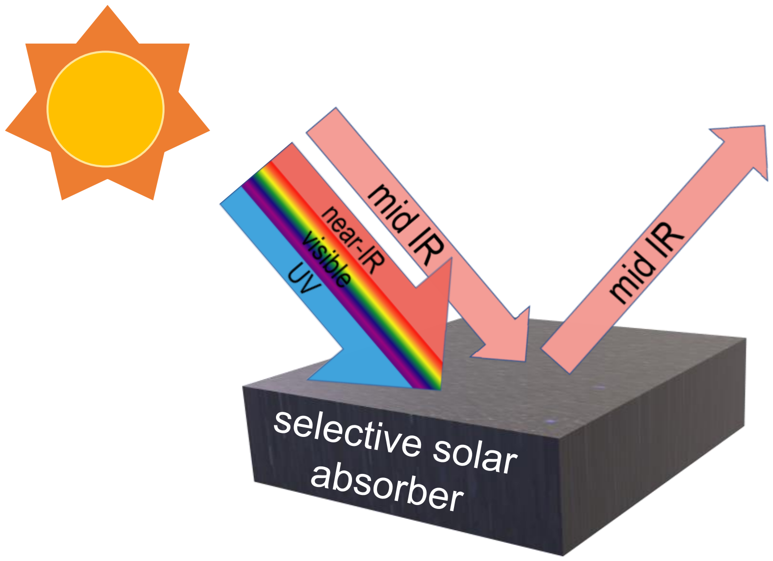

Supplementary Fig. 1. Schematic of spectrally-selective solar absorber with perfect absorption in the solar spectrum (0.3–2.5 µm) and no absorption in the mid-IR region (>2.5 µm). Reproduced and modified with permission from Ref. 1. Copyright Wiley, 2021.

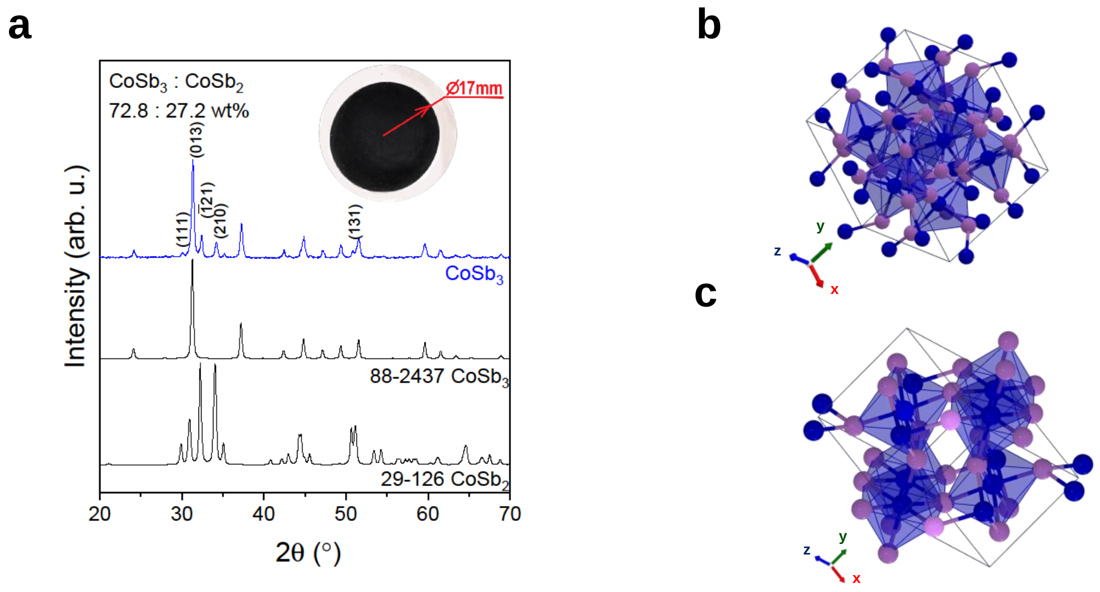

Supplementary Fig. 2. XRD Analysis and Crystal Structures of $CoSb_3$ and $CoSb_2$. a, XRD pattern of the $CoSb_3$ powder. Inset: optical photo of the $CoSb_3$ membrane after filtration. **b,** $CoSb_3$ crystal structure. **c,** $CoSb_2$ crystal structure (blue and purple balls represent Co and Sb respectively).

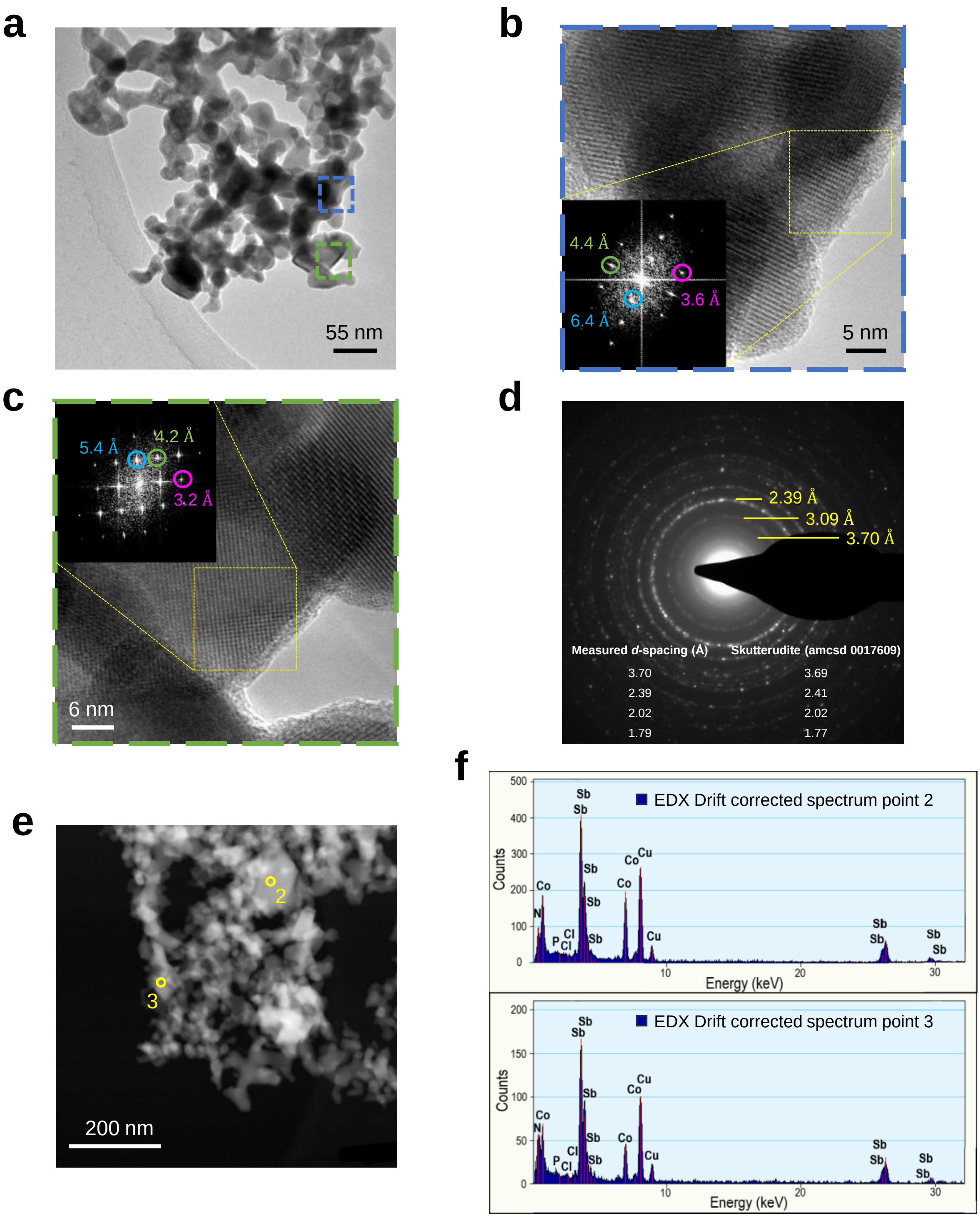

Supplementary Fig. 3. Structural characterizations of the $CoSb_3$ powder by TEM. a, TEM micrograph at low magnification of the cobalt antimonide sample. Magnified blue and green squares are presented in **b** and **c** figure, respectively**b, c,** High resolution TEM micrographs of $CoSb_3$ (b) and $CoSb_2$ (c) phase. The inserted FFT pattern corresponds to the yellow square. **d,** SAED pattern of the cobalt antimonide sample; **f,** EDS drift points analysis of the cobalt selenide sample, taking into account the HAADF-STEM micrograph (**e**) as reference.

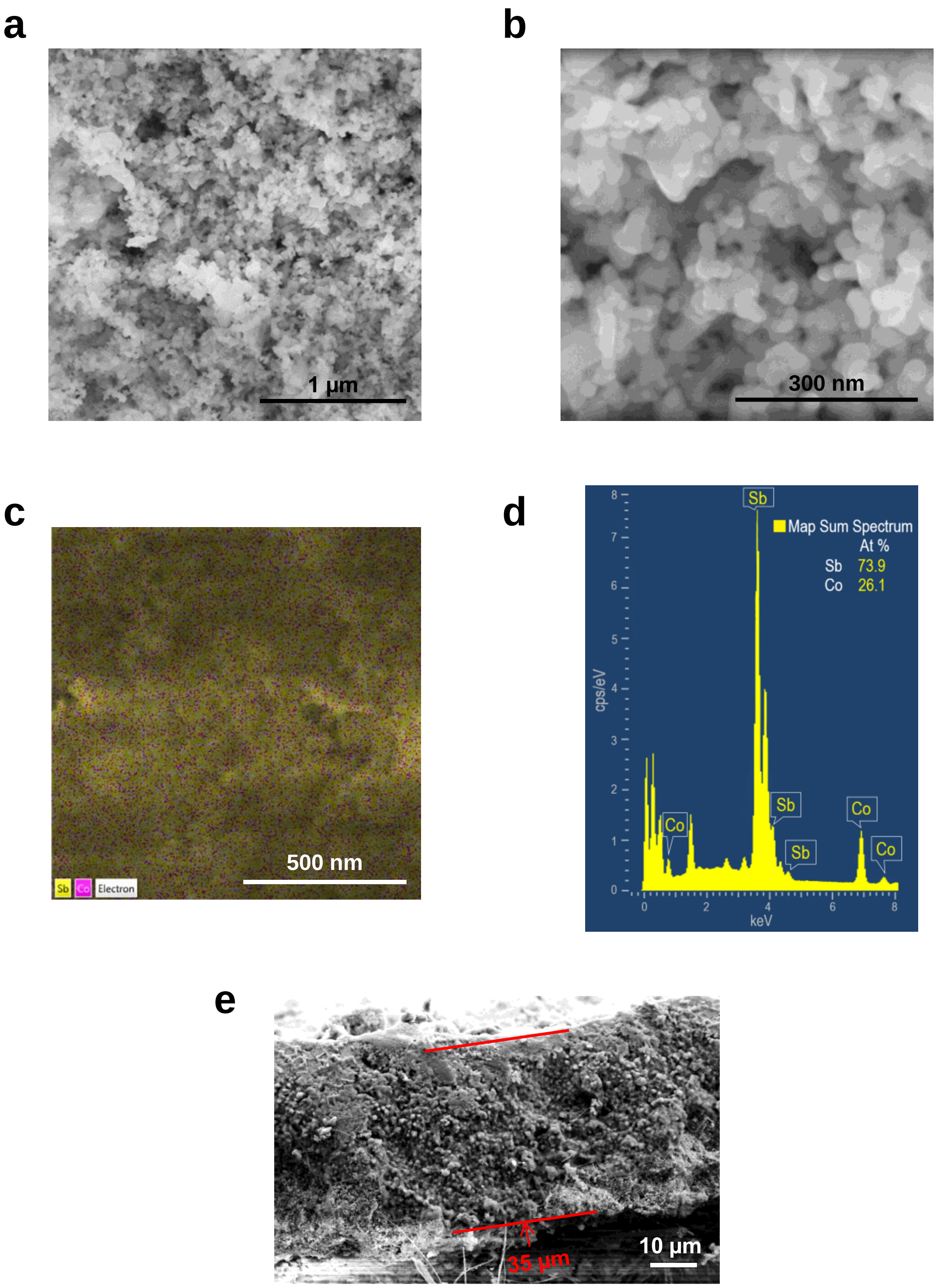

Supplementary Fig. 4. Morphology and compositional information of the $CoSb_3$ sample. a, b, Magnified SEM images. **c,** EDS elemental mapping results. **d,** Average atomic weight of EDS measurement. **e,** Cross-sectional view of $CoSb_3$ 0.1 membrane.

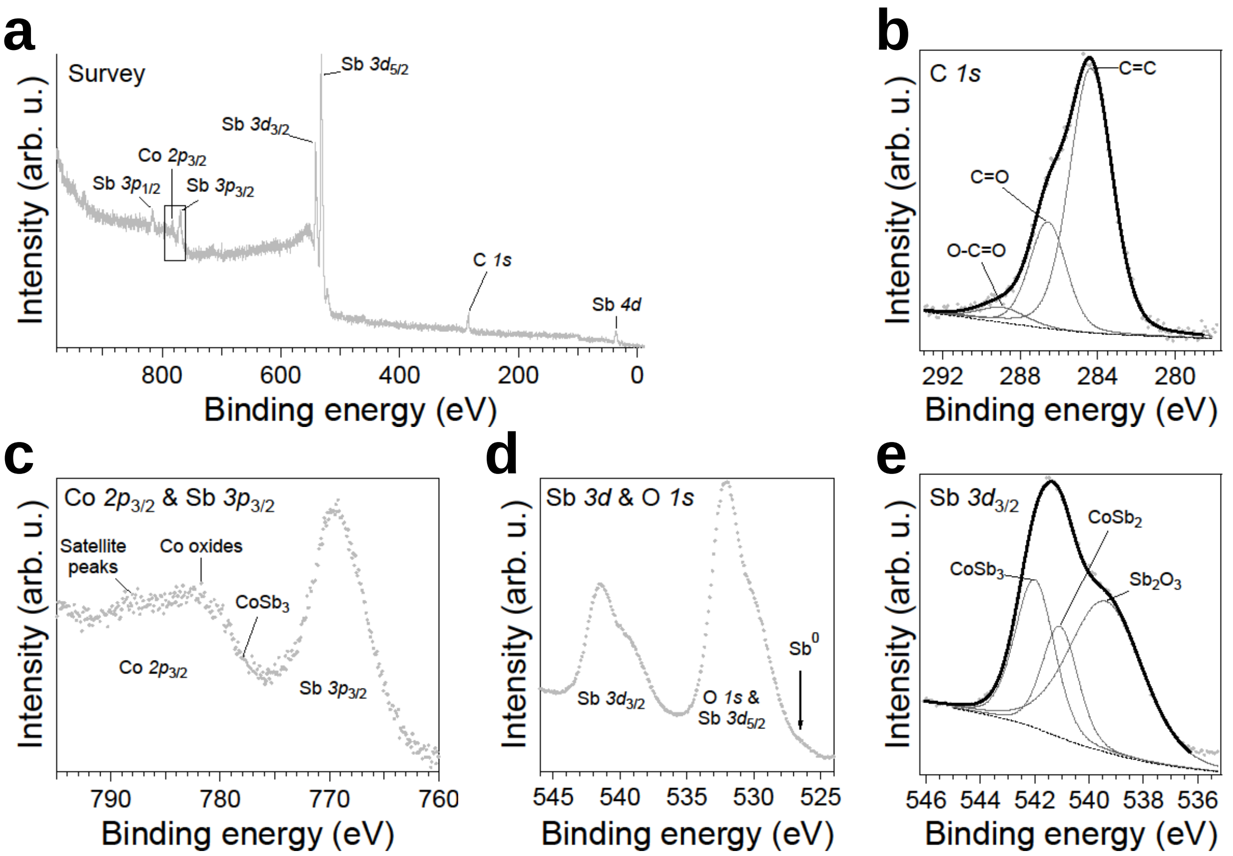

Supplementary Fig. 5. XPS spectrums of the $CoSb_3$ powder. a, XPS survey spectra. **b–e,** XPS spectra of the C1*s*, Co2$p_{3/2}$&Sb3$p_{3/2}$, Sb3*d*&O1*s*, and Sb3$d_{3/2}$ spectral regions.

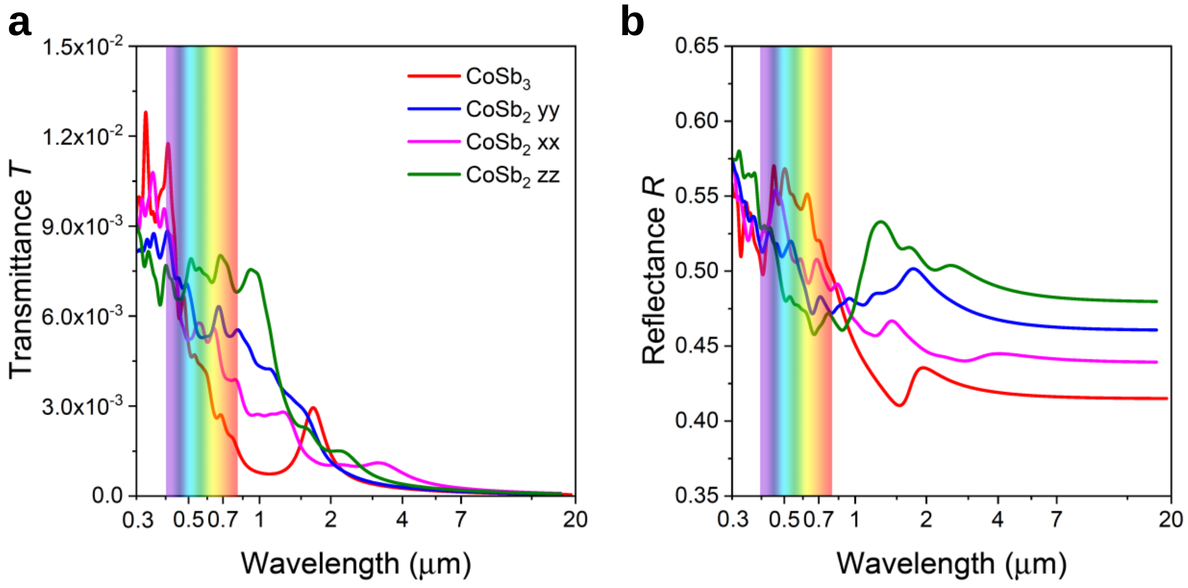

Supplementary Fig. 6. Theoretical optical characterizations of the $CoSb_2$ and $CoSb_3$. a, The normalized to 1 transmittance spectra of $CoSb_2$ and $CoSb_3$. **b,** The normalized to 1 reflectance spectra of $CoSb_2$ and $CoSb_3$.

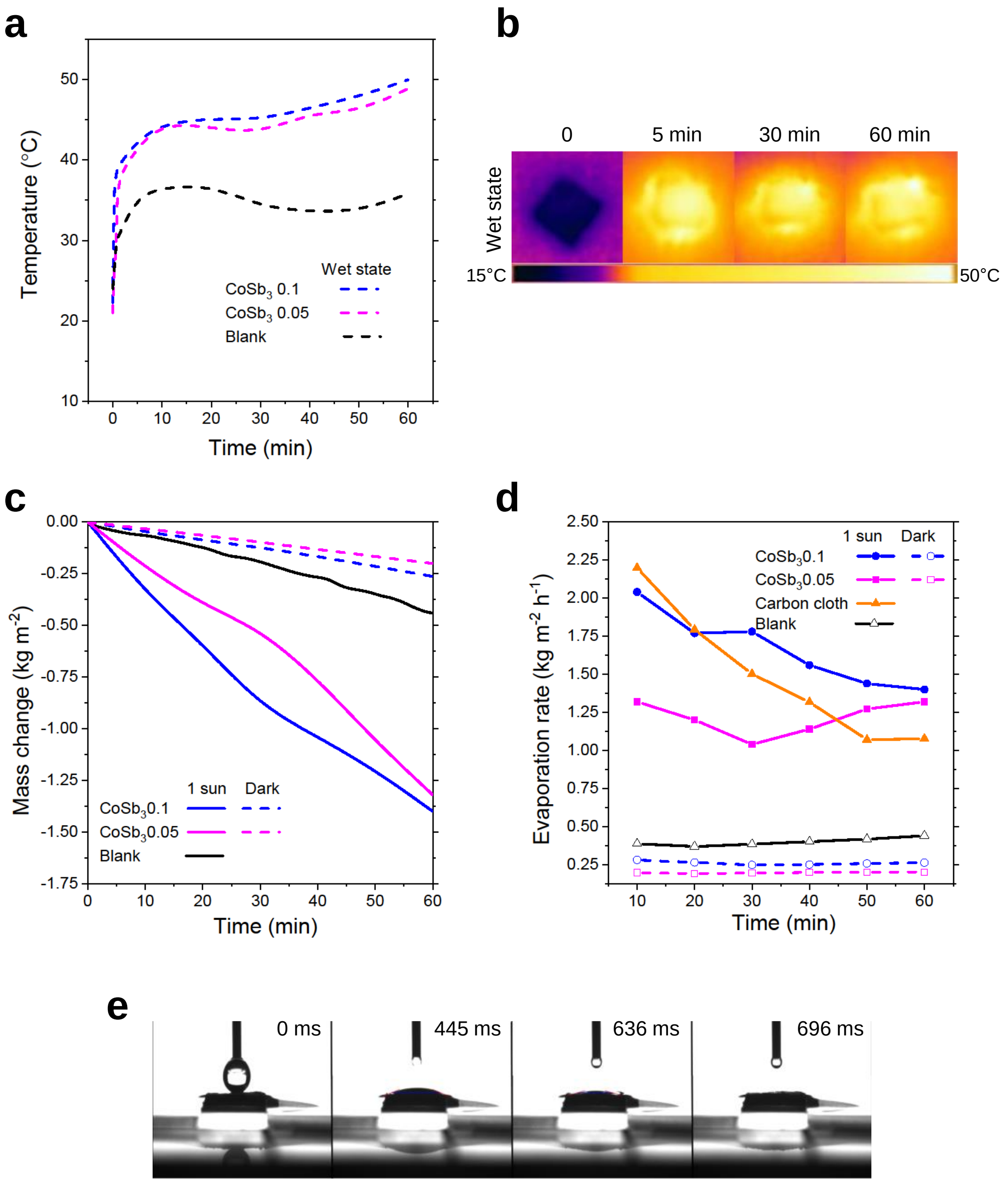

Supplementary Fig. 7. Solar water evaporation test and wettability test of $CoSb_3$ membrane. **a,** Photothermal behavior of wet $CoSb_3$ membrane under 1 sun irradiation. **b,** Time-lapse IR images of $CoSb_3$ 0.1 g membrane during water evaporation test. **c,** Curves of mass loss of water for the $CoSb_3$ membrane under 1 sun irradiation and dark condition. **d,** Evaporation efficiency of the membranes under 1 sun irradiation and dark condition. The evaporation rate obtained by applying a benchmarking carbon cloth is also reported. **e,** Time-lapse snapshots of absorbing a water droplet by the photothermal $CoSb_3$ membranes.

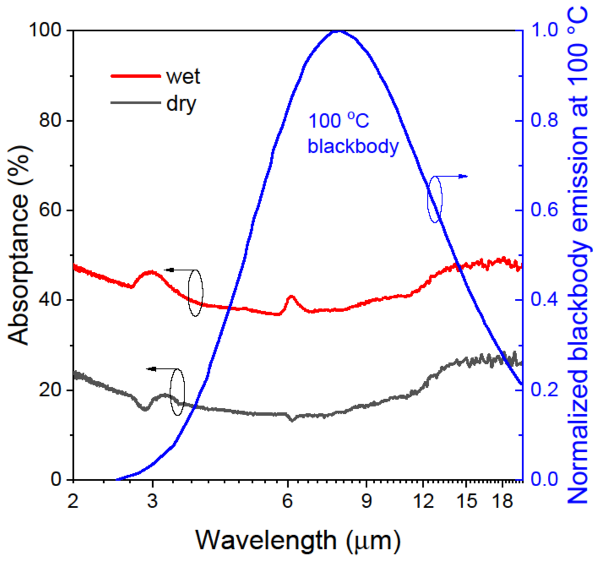

Supplementary Fig. 8. Absorption properties of the dry and wet sample in the IR region. The black body emission at 100 °C is also reported to highlight the SSA properties of the sample in both the dry and wet state.

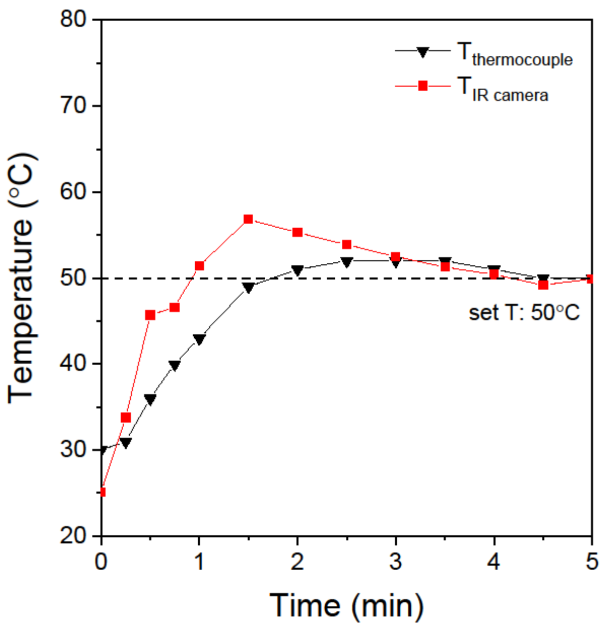

Supplementary Fig. 9. Temperature measurements from the thermocouple embedded in the hotplate and from the IR camera during the transient between room temperature and the temperature set at 50 °C. After 5 minutes a stable condition is reached, and the temperature measurement is reliable. For higher temperatures, transients up to 10 minutes were recorded.

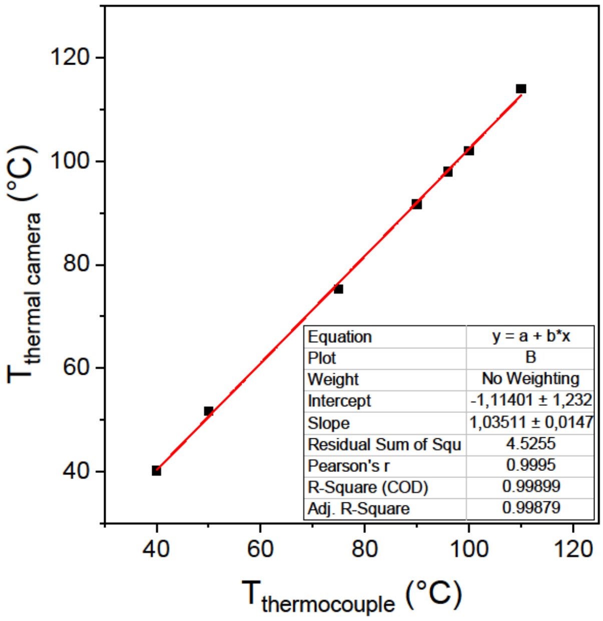

Supplementary Fig. 10. Calibration curve of the temperature measured by a thermocouple integrated into a hotplate and the thermal IR camera pointing at the surface of the hotplate.

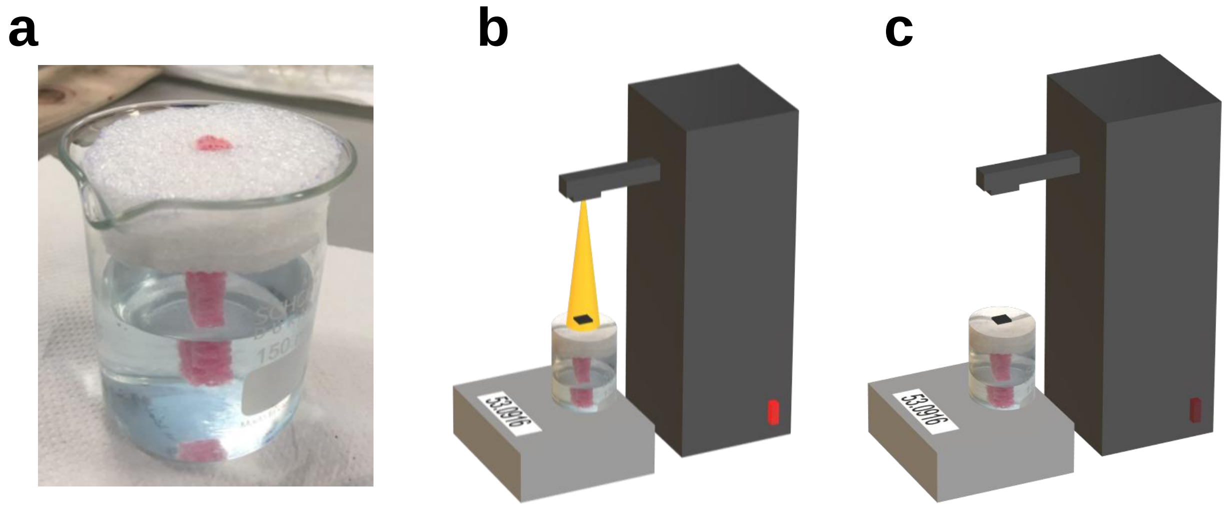

Supplementary Fig. 11. The setup for solar water evaporation test. a, The setup of the solar vapor generation measurement. **b,** The schematic illustration of solar vapor generation test under 1 sun. **c,** The schematic illustration of vapor generation test under dark conditions.

Supplementary Note 1

The crystallite size was determined by applying the Scherrer equation presented in Equation 1[2]:

$$D = \frac{0.9\lambda}{\beta \cos\theta} \qquad \text{(Eq. 1)}$$

where 0.9 – Scherrer constant, λ is the CuKα wavelength (0.154056 nm), β is the full width at half maximum of the (*hkl*) peaks at the diffraction angle 2θ. The average crystallite size *D* calculated from the strongest (013) reflection of the $CoSb_3$ phase at 31.18° is 29.2 nm, while for the $CoSb_2$ phase from the (-121) reflection the average crystallite size at 32.40° is 24.0 nm. The value is in good correlation with the SEM observation shown in Supplementary Fig. 4a.

Supplementary Note 2

Through the Rietveld refinement, the model assessment is evaluated by the blend of numerous indices, among which the residual of least-squares refinement (R_p), weighted residual (R_{wp}), experimental residual (R_{exp}) and goodness of fit (GOF) are the most demanding[3]. R_p displays the quantitative discrepancy between the observed and calculated data points, R_{wp} weights the residual so that data points with higher intensity are more important than data points with low intensity. While R_p and R_{wp} compare the calculated pattern to the experimental data, the R_{exp} evaluates the quality of the data and GOF is defined as $(R_{wp}/R_{exp})^2$. In general, the model can be accepted as correct in the case when R_p or R_{wp} is less than 10% and GOF is less than 4[4]. For the model fitting, the background was refined using a polynomial and the profile function was refined through Pseudo-Voigt function. From the Rietveld refinement the reliability factors for synthesized $CoSb_3$ are R_p = 9.91%, R_{wp} = 12.37%, R_{exp} = 6.73% and GOF = 3.37.

Supplementary Note 3

XPS data of the $CoSb_x$ powder were obtained by using a non-monochromatized Al source with the main emission line ($K_{\alpha 1,2}$) at $h\nu$ = 1486.6 eV and two secondary emission lines ($K_{\alpha 3}$ and $K_{\alpha 4}$) at $h\nu$ = 1496.4 and 1498.4 eV with approximately 9.6% intensity of the main line. The presence of secondary lines gives rise to replicas of the photoemission peaks separated by the energy difference of the emission lines (9.8 and 11.8 eV). A replica is visible on the low binding side of the Sb3$d_{5/2}$ peak in the survey spectrum Supplementary Fig. *5*5a. The XPS spectra were measured at room temperature, with the sample surface oriented at 45° with respect to the incident photon beam and normal to the analyzer axis and with a probing depth of order 1 nm. The binding energies of the photoemission peaks were calibrated with respect to Au4*f* lines of an Au reference sample.

The main features in the survey spectrum are ascribed to Sb3*p*, Co2*p*, Sb3*d*, C1*s*, and Sb4*d* core levels. From the core level spectra of C1*s* in Supplementary Fig. *5*5b, the presence of some charging effects was detected. The graphitic component of the C1*s* line was found at 286.25 eV, which is much deeper than expected. To evaluate the magnitude of the shift the binding energy value of graphitic carbon adsorbed on Sb_2O_3 (284.35 eV) was used, as the surface of the $CoSb_x$ particles presents an oxidized surface layer of Sb_2O_3[5]. A rigid shift by 1.90 eV of the C1*s* line towards lower binding energies was applied to compensate for the charging effects in all spectra.

The spectral region between 760 and 810 eV presents intense Sb3*p* lines, which partially overlap with the Co2*p* lines. Supplementary Fig. *5*5c reports the spectral region of the Co2$p_{3/2}$ and Sb3$p_{3/2}$ lines. The expected energy for the Co2$p_{3/2}$ level of $CoSb_3$, which is the prevalent compound of the $CoSb_x$ powder, is 778.0 eV[6]. However, at this energy we do not observe a clear feature ascribed to $CoSb_3$. Instead, the region between 780 and 790 eV presents peaks related to Co oxides and shake-up satellite peaks. Therefore, the analysis of the Co levels does not provide evidence for the existence of $CoSb_x$ compounds in the sample.

From the spectra of Sb3*d* in Supplementary Fig. *5*5d, the $Sb_{5/2}$ and $Sb_{3/2}$ lines are separated by 9.4 eV and present more than one component. Notably, the $Sb_{5/2}$ line overlaps with the O1*s* level, with binding energy in the range 530÷532 eV, and with the replicas of the $Sb_{3/2}$ line. A residual intensity from elemental Sb (Sb^0) can be observed on the low binding energy side of the $Sb_{5/2}$ line. With the fitting of the $Sb_{3/2}$ line in Supplementary Fig. *5*5e, 3 peaks at 541.96, 541.27, and 539.4 eV are observed. By subtracting 9.4 eV, the positions of the corresponding $Sb_{5/2}$ peaks, which are usually reported in the literature, can be found. The peak at 530.0 eV is attributed to Sb_2O_3, while 532.56 and 531.87 eV are ascribed to two different compositions of $CoSb_x$[7]. The more intense and deeper component is compatible with the presence of $CoSb_3$, while the other component could be associated to compounds with $x<3$, like $CoSb_2$.

Supplementary Note 4

The calculation procedure for investigating $CoSb_2$ and $CoSb_3$ structures was reported by Li Yang *et al.*[17]. All calculations were performed within the framework of DFT using the Vienna ab-initio simulation package (VASP)[18]. The projector-augmented wave (PAW) method was used together with a plane-wave energy cutoff of 520 eV, and the Perdew-Burke-Ernzerhow (PBE) functional was used to describe exchange-correlation effects[19]. The respective in-plane lattice parameters for $CoSb_3$ and $CoSb_2$ were optimized for the primitive unit cell, using a 2×2×2 and 3×3×3 *k*-points mesh respectively. For calculation of the optical properties, the HSE screened hybrid functional was used and the systems were converged concerning the doubled *k*-points mesh[20]. All crystal structures including the lattice parameters and atomic positions were allowed to relax until the total energy and force were converged to 10–6 eV and 0.01 eV $Å^{-1}$, respectively. The optical properties of the crystals were described by the dielectric function. Further, the VASPKIT code was used for post-processing of the VASP calculated data[21]. 'Source data are provided as a Source Data file.

Supplementary Note 5

The solar water evaporation rate was assessed with the evaporation of saline water (called seawater after), photothermal behavior of the wet $CoSb_3$ membrane is presented in Supplementary Fig. 7a, b. Under 1 sun irradiation, the surface temperature of $CoSb_3$ at AM 1.5G condition stands at 41.5 °C after 5 min of illumination. Despite the high surface temperature values under dry conditions, the surface temperature under wet conditions was on par with previous reports as shown in Supplementary Table . After 10 min the surface temperature for $CoSb_3$ 0.05 and $CoSb_3$ 0.1 reached 48.9 °C and 50 °C, respectively. The time-dependent weight change of water at 1 sun irradiation and under dark conditions was plotted in Supplementary Fig. 7c. From the slope of the curves, the membranes with a mass loading of 0.1 g showed a higher evaporation rate compared with the membranes with 0.05 g of powder, which was three times as much as the evaporation rate of bulk water under 1 sun. Supplementary Fig. 7d displays high vapor yields of 1.4 and 1.32 kg m^{-2} h^{-1} for the $CoSb_3$ 0.1 and 0.05 membrane respectively, which are close to the evaporation rate of 1.47 kg m^{-2} h^{-1} assuming 100% solar-to-vapor energy transfer efficiency under 1 sun irradiation[22]. For efficient water evaporation, minimizing heat loss is an important criterion that leads to maximizing the conversion efficiency. The absorber with lower thermal conductivity decreases of heat loss, while $CoSb_3$ owes rather high thermal conductivity (from the literature, around 4.5 W m^{-1} K^{-1} [23]). Nevertheless, the Styrofoam insulator substrate on which the absorber is placed prevent the heat losses to bulk water.

Supplemenraty Note 6

The effect of water on SSA properties of $CoSb_x$ was investigated by recording the absorption in the IR spectral region 2–20 µm. The results are reported in the Supplementary Fig. 8 below, together with the normalized black body emission at 100 °C. As expected, water induces a worsening of the SSA properties, but the sample still maintains spectral selectivity, guaranteeing low enough emissivity under solar illumination, which results in high evaporation rate.

Supplementary Note 7

Spectral absorptance α can be expressed in terms of total reflectance $R(\lambda,\theta)$ by Equation 2, and ε can be defined through α by Equation 3[11]:

$$\alpha(\lambda,\theta) = 1 - R(\lambda,\theta) \quad \text{(Eq. 2)}$$

$$\varepsilon(\lambda,T) = \alpha(\lambda,T) \quad \text{(Eq. 3)}$$

where $R(\lambda,\theta)$ is the sum of both collimated and diffuse reflectance, λ is the wavelength (μm), θ is the incidence angle of light, and T is the given temperature (K).

The spectrally averaged solar absorptance $\bar{\alpha}$ and averaged thermal emissivity $\bar{\varepsilon}$ are defined by Equations 4 and 5.

$$\bar{\alpha} = \frac{\int_{0.3\mu m}^{2.5\mu m} \alpha(\lambda) E_{solar}(\lambda) \mathrm{d}\lambda}{I_{solar}} \quad \text{(Eq. 4)}$$

$$\bar{\varepsilon}(T) = \frac{\int_{0.3\mu m}^{20\mu m} \varepsilon(\lambda) E_b(\lambda,T) \mathrm{d}\lambda}{\sigma T^4} \quad \text{(Eq. 5)}$$

where $E_{solar}(\lambda)$ is the spectral solar power (AM 1.5G), I_{solar} is the total solar power density (1 sun, 1 kW m^{-2}), $\varepsilon(\lambda)$ – the emissivity at the wavelength λ, $E_b(\lambda,T)$ – the spectral irradiance of a blackbody at T = 373.15 K, σ – the Stefan-Boltzmann constant ($5.6696 \cdot 10^{-8}$ W m^{-2} K^{-4}), T – the operating temperature (373.15 K), and T_0 is the ambient temperature (293.15 K).

Supplementary Note 8

The output temperature of the IR camera was calibrated to assure an accurate measurement of the surface temperature during the various temperature measurements reported in the study. For the purpose, the temperature measured by a thermocouple integrated in a hotplate and the temperature read by the thermal camera pointing at the surface of the hotplate were simultaneously acquired in the temperature range 40–110 °C. The investigated range was chosen to include the characteristic temperatures considered in the experiments and to guarantee the accuracy of the measurements under the different conditions. When changing the temperature set of the hotplate, a time interval ranging between 5 to 10 minutes was needed to reach a stable asymptotic temperature (see Supplementary Fig. 9). The temperature measurements of both the thermocouple and the IR camera refer to asymptotic condition, when temperature fluctuations were negligible. Supplementary Fig. 10 reports the experimental data of IR camera calibration. The experimental data are well fitted (R=0.99899) by a linear interpolation (Eq. 6):

$$T_{\text{thermal camera}} = a + b \cdot T_{\text{thermocouple}} \qquad \text{(Eq. 6)}$$

where a = (-1.1±1.2) °C and b = (1.035±0.015) °C.

These results indicate that the output of the IR camera are reliable and that the IR camera can be safely used for the measurement of the surface temperature of the samples investigated in this study.

Supplementary Note 9

For the water evaporation performance, the 30 ml seawater was simulated by 0.6 mol l^{-1} NaCl solution in the deionized water (3.5 wt% average global seawater salinity). The powders with 0.05 and 0.1 g were directly loaded on a glass wool filter membrane using the vacuum filtration approach. The membrane was cut into pieces with a size of 1 cm × 1 cm, and an expandable polyethylene foam was utilized as a thermal insulator. For water transport from the beaker to the membrane cotton with a height of 6 cm was used, and 3 cm of height was impregnated with water. The setup for the solar vapor generation test and the scheme of the experiment under 1 sun and dark conditions are presented in Supplementary Fig. 11. The mass of the water loss was measured by an electronic balance Orma BC 300 with 1 mg resolution. Simultaneously, during the experiment, the stability of the balance was tested by weighting a standard inorganic body with a mass of ~ 80 g. The evaporation mass loss of the receiver was measured under dark conditions and solar illumination 1 sun for 1 hour. Water evaporation experiments were carried out at a room temperature of (24±1) °C and air humidity of ∼ 50%. The evaporation rate E (kg m^{-2} h^{-1}) was calculated according to equation 7[41]:

$$E = \frac{\Delta m}{S \cdot \tau} \qquad \text{(Eq. 7)}$$

where Δm is the mass loss of water (kg), S is the area of the evaporation (m^2), and τ is the evaporation time (h).

Supplementary Table 1. Comparison of the optical performance of solar absorbing materials

**Material**	**Solar absorptance $\bar{\alpha}$ (%)**	**IR emissivity $\bar{\varepsilon}$ (%)**	**Spectral selectivity $\bar{\alpha}/\bar{\varepsilon}$**	**Ref**
Ag	3	<1	-	8
Al	17	<1	-	8
W	22	7	3.1	9
Stainless steel	38	13	2.9	10
Ge	90	70	1.3	11
Co_3O_4	96	71	1.4	11
SiO_2	2	75	-	12
ZrB_2	47	9	5.2	13
TiB_2	49	15	3.3	13
HfC	63	15	4.2	14
CdTe	71	37	1.9	15
Carbon-based	95	90 – 95	1	16
$Ti_3C_2T_x$ MXene	90	17	5.3	17
$CoSb_3$	96	18	5.3	This work

Supplementary Table 2. Comparison of Solar Vapor Generation Performance of Various Solar Absorbers in the seawater under 1 sun

Solar absorbers	Characterization of the solar absorber	Evaporation rate (kg m^{-2} h^{-1})	T_{wet} (°C)	T_{dry} (°C)	Ref
$Cu_{2-x}Te$	broadband plasmonic $Cu_{2-x}Te$ nanowire membrane	1.4	40	63	24
Bi_2S_3	Bi_2S_3 on the nylon membrane	1.13	63.6	-	25
CuS	plasmonic hierarchical CuS on the poly(vinylidene fluoride) membrane PVDFM	1.3	38.5	-	26
Al–Ti–O	plasmonic Al–Ti–O on the PVDFM	1.03	47.5	-	27
Pd-plasmonic wood	wood with plasmonic Pd nanoparticles	1.02	30.6	-	28
NCM	Ni particles–cellulose hybrid metamaterial	1.32	42	59	29
Ni@C@SiO	Ni@C@SiO_2 core–shell nanoparticles	1.67	31.6	-	30
AuNP/PBONF	nanoporous gold nanoparticle (AuNP)/poly(p-phenylene benzobisoxazole) nanofibre (PBONF) composite	1.42	32	-	31
DLS	double-layer structure (DLS) consisting of a carbon foam layer supporting an exfoliated graphite layer	0.97	50.1	-	32
$rGSi_{0.2}$	reduced graphene oxide film + SBA-15 on polystyrene foam	1.1	45	82.5	33
G@ZIF	nanohybrid encapsulated 2D graphene nanosheets with zeolitic imidazolate framework (ZIF) layer (G@ZIF)	1.78	50	120	34
1D-OMoSNSA-M	1D-O-doped MoS_{2-x} nanosheets	2.5	43	81	35
$Ti_3C_2T_x$	MXene	-	-	89	17
TiN	plasmonic metamaterial TiN nanoparticles covered by a polyethylene bubble wrap film	-	-	105	36
CuCrMnCoAlN	multilayered CuCrMnCoAlN-based SSA	-	-	109.6	37
HfNbTaTiZr	multilayered HfNbTaTiZr-based SSA	-	-	110	38
Ni/Y_2O_3	multilayer structure (single Ni atom on the Y_2O_3 nanosheets)/quartz/(AlN_x/Al)/vacuum/quartz	-	-	288	39
Bi_2Te_3/Cu<br>Bi_2Te_3	Multilayer structure CaF_2 glass/vacuum/Bi_2Te_3/Cu<br>Standalone Bi_2Te_3	-	-<br>-	317<br>93	40<br>39
$CoSb_3$	0.1 g of $CoSb_3$ on the glass wool membrane	1.4	50	101.7	This work

Supplementary References:

1. Li, Y., Lin, C., Huang, J., Chi, C. & Huang, B. Spectrally Selective Absorbers/Emitters for Solar Steam Generation and Radiative Cooling-Enabled Atmospheric Water Harvesting. *Glob. Challenges* **5**, 2000058 (2021).
2. He, K., Chen, N., Wang, C., Wei, L. & Chen, J. Method for Determining Crystal Grain Size by X-Ray Diffraction. *Cryst. Res. Technol.* **53**, 1–6 (2018).
3. Young, R. A. The rietveld method. 38 (1993).
4. Speakman, S. A. Precision and Accuracy-Agreement Indices in HSP (An Introduction to Rietveld Refinement using PANalytical X'Pert HighScore Plus v2. 2d). *Massachusets Inst. Technol* (2010).
5. Karikalan, N., Elavarasan, M. & Yang, T. C. K. Studies on the sonochemical polymorphism of Sb2O3 on activated graphite for the electrochemical determination of imipramine. *Ultrason. Sonochem.* **64**, 105012 (2020).
6. Zhao, W. *et al.* Multi-localization transport behaviour in bulk thermoelectric materials. *Nat. Commun.* **6**, 1–7 (2015).
7. Bala, M. *et al.* Evolution of nanostructured single-phase CoSb3 thin films by low-energy ion beam induced mixing and their thermoelectric performance. *Phys. Chem. Chem. Phys.* **19**, 24886–24895 (2017).
8. Greenberg, S. A. Low Solar Absorptance and Emittance Surfaces Utilizing. *Nasa* (1968).
9. Lampert, C. M. Coatings for enhanced photothermal energy collection I. Selective absorbers. *Solar Energy Materials* vol. 1 319–341 (1979).
10. Sibin, K. P., John, S. & Barshilia, H. C. Control of thermal emittance of stainless steel using sputtered tungsten thin films for solar thermal power applications. *Sol. Energy Mater. Sol. Cells* **133**, 1–7 (2015).
11. Kennedy, C. E. Review of Mid- to High- Temperature Solar Selective Absorber Materials. (2002).
12. Ma, H. *et al.* Multilayered SiO2/Si3N4 photonic emitter to achieve high-performance all-day radiative cooling. *Sol. Energy Mater. Sol. Cells* **212**, 110584 IF 7.26 (2020).
13. Sani, E. *et al.* Titanium diboride ceramics for solar thermal absorbers. *Sol. Energy Mater. Sol. Cells* **169**, 313–319 (2017).
14. Sani, E., Mercatelli, L., Jafrancesco, D., Sans, J. L. & Sciti, D. Ultra-high temperature ceramics for solar receivers: Spectral and high-temperature emittance characterization. *J. Eur. Opt. Soc.* **7**, (2012).
15. Benlattar, M., Oualim, E. M., Harmouchi, M., Mouhsen, A. & Belafhal, A. Radiative properties of cadmium telluride thin film as radiative cooling materials. *Opt. Commun.* **256**, 10–15 (2005).
16. Vinetsky, Y., Jambu, J., Mandler, D. & Magdassi, S. Cnt-based solar thermal coatings: Absorptance vs. emittance. *Coatings* **10**, 1-12 IF 2.86 (2020).
17. Li, Y. *et al.* 2D Ti 3 C 2 T x MXenes: Visible Black but Infrared White Materials. *Adv. Mater.* **2103054**, 2103054 (2021).
18. Kresse, G. & Hafner, J. Norm-conserving and ultrasoft pseudopotentials for first-row and transition elements. *J. Phys. Condens. Matter* **6**, 8245–8257 (1994).
19. Kresse, G. & Joubert, D. From ultrasoft pseudopotentials to the projector augmented-wave method. *Phys. Rev. B* **59**, 1758–1775 (1999).
20. Krukau, A. V., Vydrov, O. A., Izmaylov, A. F. & Scuseria, G. E. Influence of the exchange screening parameter on the performance of screened hybrid functionals. *J. Chem. Phys.* **125**, (2006).
21. Wa Xu, N., Liu, J.-C., Tang, G., Genng, V. & Wen-Tong. VASPKIT: A user-friendly interface facilitating high-throughput computing and analysis using VASP code. *Comput. Phys. Commun.* 108033 (2021).

22. Li, X. *et al.* Enhancement of Interfacial Solar Vapor Generation by Environmental Energy. *Joule* **2**, 1331–1338 (2018).

23. Tafti, M. Y., Saleemi, M., Han, L., Nong, N. V. & Toprak, M. S. On the chemical synthesis route to bulk-scale skutterudite materials. *Ceram. Int.* **42**, 5312–5318 (2016).

24. Chen, C., Liu, H., Wang, H., Zhao, Y. & Li, M. A scalable broadband plasmonic cuprous telluride nanowire-based hybrid photothermal membrane for efficient solar vapor generation. *Nano Energy* **84**, 105868 (2021).

25. Wang, Z. *et al.* Bi2S3/nylon membrane photothermal absorber with water shortage warning capability for seawater desalination. *Mater. Lett.* **286**, 129188 (2021).

26. Tao, F. *et al.* Copper Sulfide-Based Plasmonic Photothermal Membrane for High-Efficiency Solar Vapor Generation. *ACS Appl. Mater. Interfaces* **10**, 35154–35163 (2018).

27. Yi, L. *et al.* Scalable and low-cost synthesis of black amorphous Al-Ti-O nanostructure for high-efficient photothermal desalination. *Nano Energy* **41**, 600–608 (2017).

28. Zhu, M. *et al.* Plasmonic Wood for High-Efficiency Solar Steam Generation. *Adv. Energy Mater.* **8**, 1–7 (2018).

29. Yuan, Y. *et al.* A Scalable Nickel–Cellulose Hybrid Metamaterial with Broadband Light Absorption for Efficient Solar Distillation. *Adv. Mater.* **32**, 1–8 (2020).

30. Yang, F. *et al.* Ni-based Plasmonic / Magnetic Nanostructures as Efficient Light Absorbers for Steam Generation. **2006294**, 1–9 (2020).

31. Chen, M. *et al.* Plasmonic nanoparticle-embedded poly(: P -phenylene benzobisoxazole) nanofibrous composite films for solar steam generation. *Nanoscale* **10**, 6186–6193 (2018).

32. Ghasemi, H. *et al.* Solar steam generation by heat localization. *Nat. Commun.* **5**, 1–7 (2014).

33. Shi, L., Wang, Y., Zhang, L. & Wang, P. Rational design of a bi-layered reduced graphene oxide film on polystyrene foam for solar-driven interfacial water evaporation. *J. Mater. Chem. A* **5**, 16212–16219 (2017).

34. Han, X. *et al.* Intensifying Heat Using MOF-Isolated Graphene for Solar-Driven Seawater Desalination at 98% Solar-to-Thermal Efficiency. *Adv. Funct. Mater.* **2008904**, 1–7 (2021).

35. Lu, Q., Shi, W., Yang, H. & Wang, X. Nanoconfined Water-Molecule Channels for High-Yield Solar Vapor Generation under Weaker Sunlight. *Adv. Mater.* **32**, 1–7 (2020).

36. Li, Y. *et al.* Solution-Processed All-Ceramic Plasmonic Metamaterials for Efficient Solar – Thermal Conversion over 100 – 727 ° C. doi:10.1002/adma.202005074.

37. Liu, X. *et al.* Enabling Highly Enhanced Solar Thermoelectric Generator Efficiency by a CuCrMnCoAlN-Based Spectrally Selective Absorber. *ACS Appl. Mater. Interfaces* (2022) doi:10.1021/acsami.2c15215.

38. He, C. Y. *et al.* Toward high-temperature thermal tolerance in solar selective absorber coatings: choosing high entropy ceramic HfNbTaTiZrN. *J. Mater. Chem. A* **9**, 21270–21280 (2021).

39. Li, Y. *et al.* Selective light absorber-assisted single nickel atom catalysts for ambient sunlight-driven CO2 methanation. *Nat. Commun.* **10**, 1–9 (2019).

40. Li, Y. *et al.* General heterostructure strategy of photothermal materials for scalable solar-heating hydrogen production without the consumption of artificial energy. *Nat. Commun.* **13**, 1–10 (2022).

41. Kim, S., Tahir, Z., Rashid, M. U., Jang, J. I. & Kim, Y. S. Highly Efficient Solar Vapor Generation via a Simple Morphological Alteration of TiO2Films Grown on a Glassy Carbon Foam. *ACS Appl. Mater. Interfaces* **13**, 50911–50919 (2021).
